# Cognitive-enhancing effects of aripiprazole: a case report

**DOI:** 10.1186/1745-0179-4-24

**Published:** 2008-10-29

**Authors:** Armida Mucci, Giuseppe Piegari, Silvana Galderisi

**Affiliations:** 1 Department of Psychiatry, University of Naples SUN, Largo Madonna delle Grazie, 80138 Naples, Italy

## Abstract

Patients with schizophrenia often present mild to severe cognitive deficits which contribute to their social disability. Second-generation antipsychotics have shown only mild to moderate beneficial effects on cognition. The present case report suggests cognitive enhancing effects of aripiprazole, a dopamine partial agonist, shown to increase dopamine release in prefrontal cortex in animal studies.

The patient was in his first-episode of schizophrenia, and had no previous exposure to first-generation antipsychotics. Before schizophrenia onset his cognitive functioning was poor and he could not attend regular courses to reach his high school degree; he started but was not able to attend the University courses for several years.

After schizophrenia onset, he was treated, in sequence, with olanzapine, amisulpride and aripiprazole. During treatment with the first two second-generation antipsychotics, positive symptoms markedly improved while cognitive functioning remained poor. During treatment with aripiprazole, clinical remission was obtained and the patient was able to attend university courses and pass several examinations. Social functioning was markedly improved.

Aripiprazole demonstrated cognitive enhancing effects in this patient. These effects were long-lasting and paralleled by a positive impact on social functioning.

## Background

Patients with schizophrenia often present mild to severe cognitive deficits which contribute to their social disability [1,2]. These deficits are present during the first episode of the illness and often persist after remission of acute psychotic symptoms. First-generation antipsychotics (FGAs) do not alleviate cognitive dysfunctions and might have a detrimental effect on cognition through the induction of extrapyramidal side effects (EPS). Second-generation antipsychotics (SGAs) have shown beneficial effects on cognition, but it is not clear whether these effects reflect a direct action of the drugs or are secondary to the reduction of EPS [3,5]. Aripiprazole, a dopamine partial agonist, is among the drugs under investigation as potential cognitive-enhancers. Its potential favorable impact on cognition might be related to an increased release of dopamine in prefrontal cortex and hippocampus, demonstrated in animal studies [6]. An open label study [7] seems to suggest a beneficial effect of aripiprazole on some aspects of cognitive functioning in patients with schizophrenia, in comparison with olanzapine. However, a true cognitive-enhancing effect has never been demonstrated in humans.

We describe hereafter the case of a patient in his first-episode of schizophrenia, in which cognitive-enhancing effects of aripiprazole were observed.

## Case presentation

When the patient was first seen at the Center for Psychotic Disorders of our University Department of Psychiatry, he was a 27 year-old man in his first episode of schizophrenia (undifferentiated subtype). The onset of schizophrenia was estimated at 26 years of age. He had experienced school difficulties and could not attend regular courses to reach his high school degree. He started but was not able to attend the University courses for several years before schizophrenia onset.

He had been never treated with FGAs and was experiencing an acute psychotic relapse two months after the interruption of treatment with olanzapine (10 mg/die).

According to his parents and the referring doctor, he had responded well to olanzapine, therefore, the drug was reintroduced (10 mg/die) while starting cognitive psychotherapy to increase motivation to treatment. Acute psychotic symptoms and active withdrawal improved in 1 month; however, he presented difficulty in concentrating and attention deficits. In the following 5 months, positive symptoms markedly improved (46% reduction of the pre-drug Positive and Negative Symptoms Scale (PANSS) positive factor score), but the patient experienced excessive weight increase (about 10 Kg with respect to pre-drug period) and poor response of cognitive dysfunctions (23% reduction of PANSS scores on the cognitive factor), as well as of social disability (Social and Occupational Functioning Assessment Scale (SOFAS) = 51). He could not persist in either work or study activities for more than a few days. He dropped from drug treatment after 10 months. He experienced an acute psychotic relapse and gave his consent to restart an antipsychotic drug after 2 months. Due to the limited effect of olanzapine on cognitive dysfunctions, the patient was started on amisulpride (gradually titrated to 600 mg/die). Psychotic symptoms markedly improved in 6 weeks, while cognitive dysfunctions presented a modest amelioration: he continued to spend most of the morning hours in bed, experienced learning deficits and could not study. After 6 months on amisulpride, he showed a marked improvement on PANSS positive factor (with 48% reduction of the pre-drug score) and a modest improvement on the cognitive factors (25% reduction of pre-drug score). His weight did not return to baseline levels, although no further increase was observed. After 6 months of treatment, the patient was shifted to aripiprazole (10 mg/die). In 3 months of treatment with the latter drug he attained full clinical remission. PANSS positive, negative and cognitive factors showed marked improvement (pre-drug score reduction of 61%, 43% and 64%, respectively). On SOFAS the patient reached the score of 70. During the first 10 months of treatment, he returned to his baseline weight. He was able to attend several university courses, to study and learn with regularity, and in 1 year of treatment he could pass 6 exams. He was feeling "..normal" and willing "...to manage life without drugs". He decided to stop medication without doctor's consent. In the following two months, he experienced a relapse and gave his consent to restart aripiprazole. The drug was reintroduced at 10 mg/die, but it was deemed appropriate to increase the dosage at 15 mg/die after 3 weeks. Clinical remission and a marked improvement of cognitive dysfunctions were attained in 4 months. He was able to attend 3 courses regularly and to pass one exam during the first 5 months of treatment. He could make new friendships among university students and started to attend a political group. After 6 months of treatment with aripiprazole, PANSS positive, negative and cognitive factors attained the largest improvement (reduction of pre-drug scores of 60%, 59% and 57%, respectively) and the SOFAS score increased to 80. He gained about 3 Kg during the first 2 months and then stabilized his weight.

## Discussion

In our opinion this case report suggests remarkable effects of aripiprazole on ecological, real-life indicators of cognitive functions, i.e., the ability to attend University courses and to pass formal examinations, in a patient who, before schizophrenia onset, presented poor cognitive functioning and could not attend regular courses to reach his high school degree. Aripiprazole also determined a marked improvement of psychosocial functioning, as assessed by SOFAS score of 80. These results were observed with aripiprazole but not with either olanzapine or amisulpride, in spite of the positive effects of these two antipsychotic drugs on both positive and negative symptoms.

SGAs have shown beneficial effects on cognition [3,5,7-9], and hypothesized mechanisms include a lower propensity, with respect to FGAs, to induce cognitive blunting due to a low affinity for the dopamine D2 receptor, an action on the 5-HT(1A) receptor, which promotes a release of dopamine in prefrontal cortex, as well as through neuroprotective action [5,10,11]. Aripiprazole might have a cognitive-enhancing effects through the last two of the previous mechanisms, as well as through an increase of dopamine transmission in prefrontal cortex and hippocampus [6].

## Conclusion

Aripiprazole demonstrated cognitive enhancing effects in this patient. These effects were long-lasting and paralleled by a positive impact on social functioning.

## Consent

The patient was informed about the nature and aims of the present case report, and a draft containing all information about his history and therapy to be included in the report was provided to him for comments and queries. After his approval of the draft, a written informed consent was obtained.

## Competing interests

The authors received fees for educational programmes (AM, SG and PG) or advisory boards (SG) by AstraZeneca, Bristol-Myers Squibb, Janssen-Cilag and Innova-Pharma.

## Authors' contributions

AM and PG conceived and drafted the manuscript; SG helped to draft the manuscript, revising it critically for important intellectual content. AM was in charge of the patient and PG carried out the clinical evaluations. All authors read and approved the final manuscript.
